# Psychosocial Factors Affecting the Association between a Healthy Lifestyle Behavior Intervention and Depressive Symptoms in Low-Income Overweight or Obese Mothers with Young Children: A Mediational Analysis

**DOI:** 10.26502/jppch.74050090

**Published:** 2022-01-05

**Authors:** Lorraine B Robbins, Mei-Wei Chang, Jiying Ling, Roger Brown

**Affiliations:** 1 College of Nursing, Michigan State University, MI, United States; 2 College of Nursing, The Ohio State University, OH, USA; 3 School of Nursing, University of Wisconsin-Madison, WI, United States

**Keywords:** Coping, Self-efficacy, Maternal Depression, Motivation, Women

## Abstract

**Background:**

Depressive symptoms are particularly prevalent among low-income overweight or obese mothers with young children, indicating the importance of understanding and addressing this serious health condition. Although lifestyle behavior interventions are promising for alleviating depressive symptoms in low-income overweight or obese mothers with young children, mechanisms underlying the association between these interventions and depressive symptoms in this priority population remain unknown.

**Objective:**

A secondary analysis of data from a randomized controlled trial that tested a 16-week community-based lifestyle behavior intervention for low-income overweight or obese mothers with young children was conducted to examine whether autonomous motivation, coping self-efficacy, and emotional coping mediated the association between the intervention and depressive symptoms.

**Methods:**

The analysis included 338 participants who completed data collection at baseline and immediately after the intervention. Participants responded to validated surveys measuring autonomous motivation, coping self-efficacy, emotional coping, and depressive symptoms. To test mediation effects, composite indicator structural equation modeling was performed adjusting for baseline measures. The proportion of maximum possible (POMP) scores in the outcome variable per unit change in the predictor variables was used to calculate effect size.

**Results:**

The intervention alleviated depressive symptoms (*B* = −2.42, *p* = 0.015, POMP = −4.31%), and coping self-efficacy negatively and fully mediated the association between the intervention and depressive symptoms (*B* = −1.42, *p* = 0.002, POMP = −2.53%). Autonomous motivation and emotional coping were not significant mediators.

**Conclusions:**

Interventions aimed to alleviate depressive symptoms in low-income overweight or obese mothers with young children should include strategies to improve coping self-efficacy. However, continued research is needed to identify other mechanisms that may be contributing to the effect of lifestyle behavior interventions on depressive symptoms in this at-risk group. This information can then be used to simplify and strengthen the interventions and potentially lead to effective dissemination and implementation.

## 1. Introduction

Maternal depression negatively impacts women’s health, work, family, and social functioning [1] and adversely affects their children’s well-being. For example, children of mothers with depression are at risk for developing emotional, cognitive, behavioral, and physical problems that may continue to affect them later in life [2]. Maternal depression and obesity are related, but the relationship between the two is complex and not well understood [3]. Regardless, obesity increases women’s risk of developing Type 2 diabetes and cardiovascular disease [4]. Therefore, the combined effect of depression and obesity results in an even greater risk of women having negative outcomes [3]. Depressive symptoms are particularly prevalent in low-income overweight or obese mothers with young children, indicating the urgency of focusing on this priority population to understand and address the problem [5, 6]. Growing evidence has shown that depressive symptoms can continue across several years after giving birth among overweight or obese mothers [7, 8]. Specifically, Lee et al. [7] noted that low-income overweight or obese mothers who reported clinically significant levels of depressive symptoms during the early postpartum period continued to experience similar depressive symptoms two years later. Horwitz and colleagues [9] also noted that mothers having young children (11–42 months of age), especially mothers who were overweight or obese and had a low income, reported a high level of depressive symptoms and were likely to continue to report the same severe depressive symptoms at a follow-up assessment occurring when their child reached kindergarten. Moreover, Woolhouse and others [10] found that the prevalence of depressive symptoms among low-income mothers was much higher at 4-year than 1-year postpartum. Collectively, this information strongly underscores the need to move beyond the early postpartum period to help low-income overweight or obese mothers with young children alleviate depressive symptoms. Yet, interventions aiming to alleviate depressive symptoms have primarily targeted mothers in the early postpartum period, resulting in a serious under-representation of low-income overweight or obese mothers with young children in research [9].

One recent systematic review and meta-analysis focusing on overweight or obese women of reproductive age showed that healthy lifestyle behavior interventions can reduce depressive symptoms in this group [11]. However, the researchers did not examine the mediating mechanisms underlying the reduction in depressive symptoms in the healthy lifestyle behavior intervention studies that were included in the systematic review and meta-analysis. Understanding these mechanisms, particularly those that are modifiable (e.g., psychosocial factors), is critically important for designing effective intervenetions to alleviate depressive symptoms [2] in the priority population. Some empirical research indicates that coping self-efficacy is negatively related to depresssive symptoms among mothers, but the relationship between emotional coping and depressive symptoms is less clear. In a cross-sectional study conducted in in Malaysia with postnatal mothers who were 7 to 42 days post-delivery, Norliza and colleagues [12] found a significant negative relationship between problem-focused coping strategies and level of depression, but the association between emotional coping strategies and depression was not significant. However, in another study, when active emotional coping was combined with both active cognitive and behavioral coping to form an active coping strategies global score, active coping strategies emerged as being negatively associated with mother’s postpartum depressive symptoms at 8 weeks [13]. In a study involving mothers of children with autism spectrum disorder, positive coping, which included positive reframing, planning, and using active or problem-focused coping strategies, mediated the association between optimism and depressive symptoms. The mothers who were more optimistic used more positive forms of coping that, in turn, reduced depressive symptoms [14]. Recently, Azale and others [15] noted that the literature is devoid of studies that examine whether positive coping strategies can alleviate perinatal depression. Studies that are longitudinal and involve interventions are particularly needed.

Both the theoretical and empirical literature suggest that autonomous motivation may also be negatively related to depressive symptoms among mothers. According to Self-Determination Theory [16], individuals satisfy their need for autonomy more when they act based on internal motives or personal perceptions that an activity is important rather than because they want to receive rewards and avoid being punished or feeling guilty or anxious. Gauthier and colleagues [17] found that autonomous motivation regarding the decision to become pregnant was negatively related to unhappiness; and the higher the autonomous motivation regarding the parental role during the latter two trimesters of pregnancy, the less mothers reported depressive symptoms two months after their child’s birth. Despite some evidence supporting a negative relationship between autonomous motivation and depressive symptoms during the transition to motherhood and the potential for interventions to increase autonomous motivation among mothers, no study was found that examined the mediating effect of autonomous motivation on the relationship between an intervention and depressive symptoms in at-risk mothers with young children.

To address the identified gaps in knowledge, a secondary analysis of data from a randomized controlled trial testing a 16-week community-based lifestyle behavior intervention was conducted to determine whether coping self-efficacy, emotional coping, and autonomous motivation mediated the effect of the intervention on depressive symptoms in the priority population. Data were collected from low-income overweight or obese mothers with young children at baseline and immediately after the intervention and then used for the analysis. Briefly, the intervention, which was designed by Chang and colleagues [18], had two components: (1) watching 10 20-minute culturally sensitive intervention videos at home and (2) joining 10 30-minute peer support group teleconferences led by trained moderators. The intervention videos, which were delivered in DVD format, featured four mothers from the priority population who demonstrated practical strategies to assist the low-income overweight or obese mothers with young children in managing stress, eating healthy, and attaining physical activity. In addition, the featured mothers shared ways to respond effecttively to negative emotions. The videos aimed to improve coping self-efficacy (perceived confidence or ability to cope effectively with challenges or manage problems) and emotional coping (use of emotional support), both of which are key concepts of Social Cognitive Theory [19]. The peer support group teleconferences included motivational interviewing techniques to increase autonomous motivation [20] or the personal reasons for engaging in health-promoting behaviors, a key concept of Self-Determination Theory [16]. Additional information about the trial protocol, procedures, and intervention have been published [18].

Recently, Chang and colleagues [21] reported intervention effects for several psychosocial variables. Compared to a comparison group, the intervention group reported significantly fewer depressive symptoms (d = -0.27) and higher coping self-efficacy (Cohen’s *d* = 0.53) and emotional coping (*d* = 0.39) at the end of the 16-week lifestyle intervention. Autonomous motivation measured at baseline and immediately after the 16-week intervention was not analyzed by Chang et al. [21].

### 1.1 Aims

This paper reports results of a new analysis examining whether autonomous motivation, coping self-efficacy, and emotional coping mediated the association between the 16-week lifestyle behavior intervention and depressive symptoms among socioeconomically disadvantaged overweight or obesity women with young children. Although this group is at high risk for depression, they are not typically included in randomized controlled trials testing interventions to alleviate the problem [22]. We hypothesized that the intervention would increase autonomous motivation, coping self-efficacy, and emotional coping; and then translate into an alleviation of depressive symptoms.

## 2. Methods

### 2.1 Participants and setting

Women were recruited from the Michigan Special Supplemental Nutrition Program for Women, Infants, and Children [23] while waiting for appointments. WIC serves individuals with an annual household income at or below 185% of the U.S. federal poverty line. From 2012–2015, trained peer recruiters invited women to be screened and then measured their height and weight (to compute body mass index [BMI]). To be eligible to participate, women had to have a BMI of 25.0–39.9 kg/m^2^, be non-Hispanic Black or White, be between 18–39 years old, and have a biological child between 6 weeks and 4.5 years old. All qualified women provided verbal and written informed consent prior to participation. The study procedure was approved by the Michigan State University and Michigan Department of Community Health Institutional Review Boards.

### 2.2 Measures

At the screening session, women provided demographic data via a paper-and-pencil survey. They self-reported their race/ethnicity, education, employment, and smoking status. They also provided their and their youngest child’s date of birth, which were used to calculate maternal age and the post-birth period, respectively. Trained interviewers, who were unaware of the participants’ randomization assignment, conducted phone interviews to collect survey data on autonomous motivation, coping self-efficacy, emotional coping, and depressive symptoms. The interviewers entered the data directly into a computer collection system. Autonomous motivation was assessed via the Treatment Self-Regulation Questionnaire (six items). Construct validity for the questionnaire has been reported [24]. Women were asked about their personal reasons for engaging in health-promoting behaviors, including managing stress, in the past three months. One item example was, “because I want to take responsibility for my own health.” Response choices ranged from 1 (not at all true) to 7 (very true). To create an autonomous motivation score, the questionnaire’s six items were averaged. A higher mean score indicated greater autonomous motivation for engaging in health-promoting behaviors. In the present study, Cronbach’s α = 0.83 at T1 and 0.84 at T2.

Coping self-efficacy was measured using a 10-item survey with reported construct validity and reliability [18]. An example of a survey item was “In the past three months, how confident are you that you can relax, even when you have too much to do?” Response choices ranged from 1 (not at all confident) to 4 (very confident). To create a coping self-efficacy score, the 10 items were averaged. A higher mean score indicated greater coping self-efficacy. In the present study, Cronbach’s α = 0.80 at T1 and 0.88 at T2. Emotional coping was measured with a 5-item survey that has established construct validity and reliability [18]. An example of a survey item was “In the past three months, how often do you deal with or prevent stress by talking to family members?” Response choices ranged from 1 (rarely or never) to 4 (usually or always). To create a coping self-efficacy score, the 5 items were averaged. A higher mean score indicated greater emotional coping. In this study, Cronbach’s α = 0.60 at T1 and 0.62 at T2. Depressive symptoms were assessed via the widely used 20-item Center for Epidemiologic Studies Depression Scale (CES-D). Concurrent validity and reliability (Cronbach’s alpha α = 0.85 – 0.90) for the scale have been reported [25]. Response choices ranged from 0 (rarely or none of the time) to 3 (most or all of the time). To create a depressive symptom score, the scores for the 20 items were summed. A higher sum score indicated more depressive symptoms [25].

### 2.3 Statistical analysis

**Figure 1:**
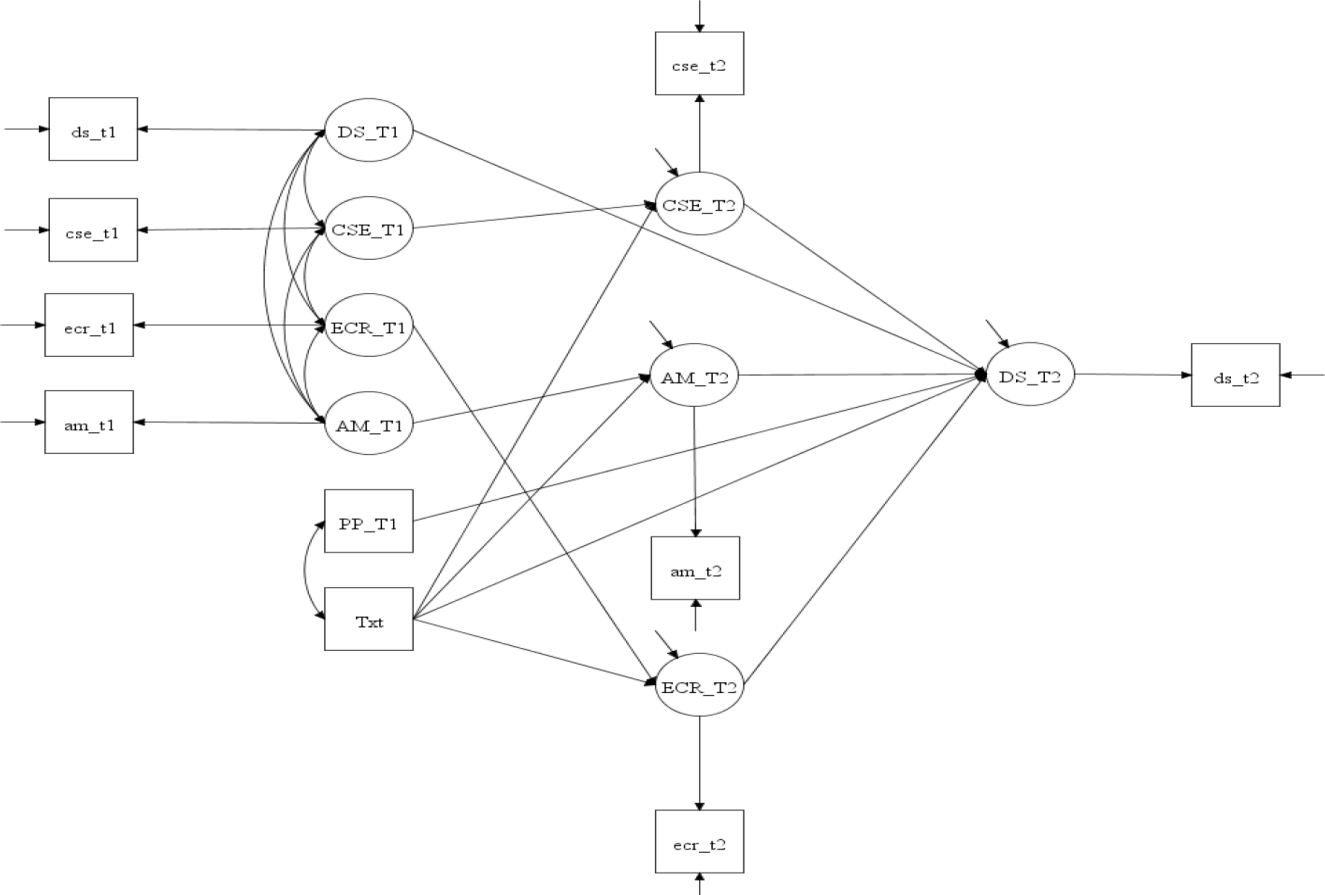
Structure of mediation model testing. Note: CSE = coping self-efficacy; ECR = emotional coping; AM = autonomous motivation; Txt = intervention; DS = depressive symptoms; PP = postpartum period (a covariate); T1 = baseline; T2 = immediately after the 16-week intervention

Mplus (version 8) was used to conduct the statistical analysis. The analysis included women (N = 338) who completed both data collection over the phone at baseline (T1) and immediately after the 16-week intervention (T2). Descriptive analysis was performed. Chi-square and independent t-test were applied to examine group differences (intervention vs. comparison) in the demographic categorical and continuous variables, respectively. Composite Indicator Structural Equation (CISE) modeling using maximum likelihood estimation [26] was performed to examine mediation effects while adjusting for baseline measurements and postpartum status. Postpartum status was used as a covariate because of its reported association with depressive symptoms [27]. As shown in Figure 1, the mediation model tested a system with four equations simultaneously: one outcome variable (depressive symptoms) and three mediators (autonomous motivation, coping self-efficacy, and emotional coping).

CISE modeling is an errors-in-variables regression approach that models measurement errors in both exogenous (predictor) and endogenous (outcome) variables [28]. CISE modeling creates latent variables by combining items of each separate measurement domain into a single indicator [26]. To control for measurement errors in a CISE model, the error variance of the indicator was fixed at (1 – α)*σ^2^ where a was the Cronbach’s alpha, and σ^2^ was the variance of the composite variable [29]. Model fit was evaluated by the R-squared value of the endogenous variable. Proportion of maximum possible (POMP) scores in the outcome variable with per unit change in the predictor variable were calculated to assess effect size: [parameter estimate/ (maximum scale value - minimum scale value)+1)]* 100 [30]. A two-tailed p-value < 0.05 was considered as statistically significant.

## 3. Results

### 3.1 Participant characteristics

Table 1 presents the demographic characteristics of the study participants (*N* = 338; n = 212 intervention; n = 126 comparison). No significant group differences in race, age, smoking and education emerged. However, women in the comparison group were at a later postpartum period and were less likely be homemakers than those in the intervention group.

### 3.2 Total and direct effects

The intervention significantly alleviated depressive symptoms (*B* = −2.42, *p* = 0.015, POMP = −4.31%). While the intervention significantly increased coping self-efficacy (*B* = 0.30, *p* < 0.001, POMP = 7.4%) and emotional coping (*B* = 0.15, *p* = 0.003, POMP = 3.81%), it did not significantly increase autonomous motivation. Autonomous motivation was signifycantly and positively associated with depressive symptoms (*B* = 1.70, *p* = 0.017, POMP = 3.02%), and coping self-efficacy was significantly and negatively associated with depressive symptoms (*B* = −4.8, *p* < 0.001, POMP = −8.56 %), but emotional coping was not significantly associated with depressive symptoms.

### 3.3 Indirect (mediation) effects

**Figure 2:**
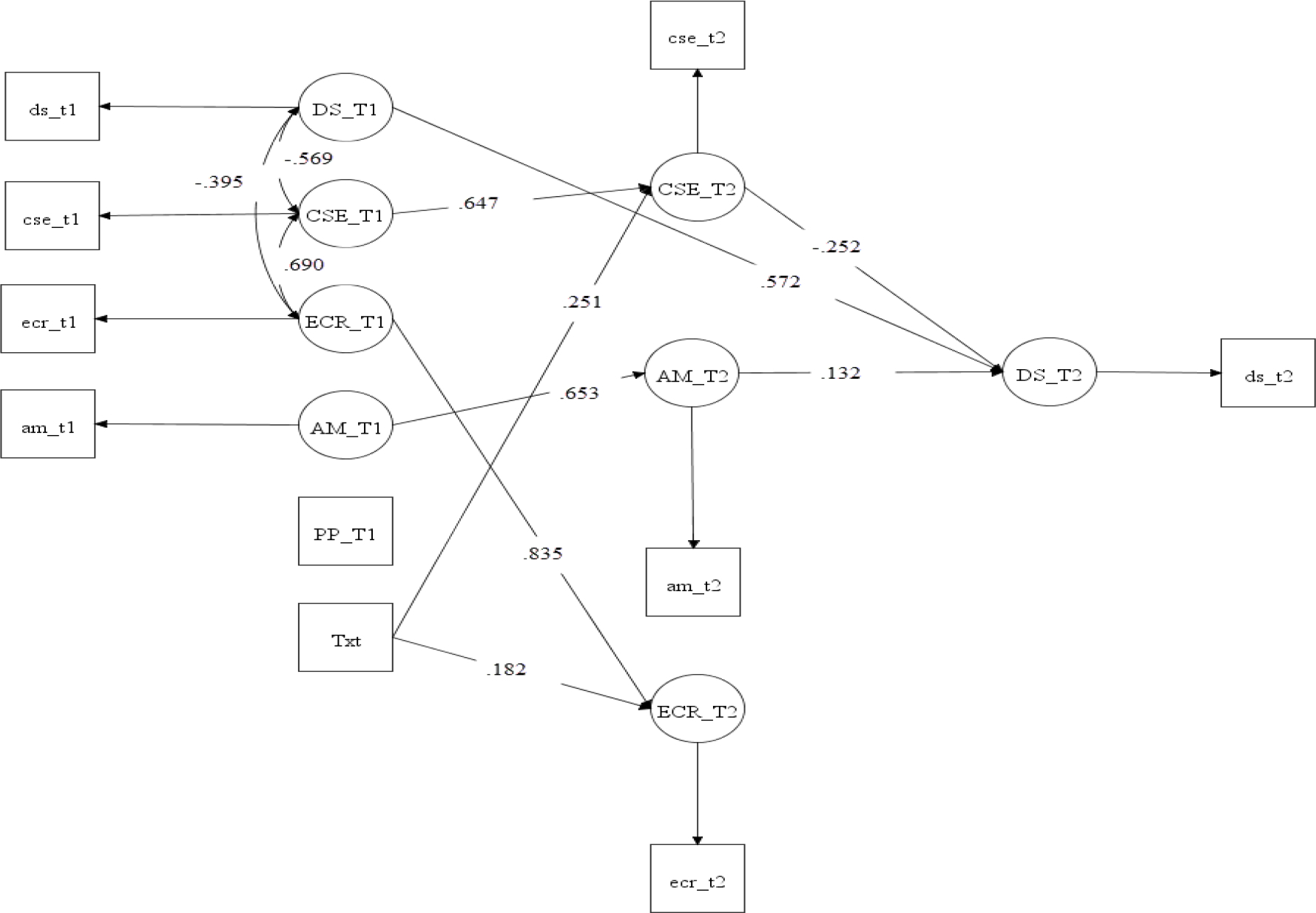
Significant paths of mediation model adjusting for baseline measures and covariate. Note: CSE = coping self-efficacy; ECR = emotional coping; AM = autonomous motivation; Txt = intervention; DS = depressive symptoms; PP = postpartum period (a covariate); T1 = baseline; T2 = immediately after the 16-week intervention. Standardized path coefficients are presented.

When assessing the potential role of autonomous motivation, emotional coping, and coping self-efficacy as mediators, neither autonomous motivation nor emotional coping was found to be a significant mediator; however, coping self-efficacy significantly and negatively mediated the association between the intervention and depressive symptoms (*B* = −1.42, *p* = 0.002, POMP = −2.53%). Additionally, after controlling for the indirect effects through autonomous motivation, coping self-efficacy, and emotional coping, the intervention had no significant influence on depressive symptoms. These results indicate that coping self-efficacy fully mediated the association between the intervention and depressive symptoms. The mediation model explained about 57% of the variance in depressive symptoms. Table 2 presents the results of direct and indirect effects while adjusting for baseline measures and the post-birth period. Figure 2 presents the significant direct and indirect paths of the mediation model.

## 4. Discussion

To our knowledge, this study is the first to examine whether autonomous motivation, coping self-efficacy, and emotional coping mediated the association between a lifestyle behavior intervention and depressive symptoms in low-income overweight or obese mothers with young children. The study findings only partially supported our hypothesis because coping self-efficacy, but not autonomous motivation or emotional coping, fully mediated the association between the intervention and depressive symptoms. In other words, coping self-efficacy emerged as the only psychosocial variable influencing the association between the intervention and depressive symptoms.

Consistent with results of a recent systematic review and meta-analysis [11], findings from the present study showed that a lifestyle behavior intervention can alleviate depression. Also, the study results showed that the intervention effectively boosted intervention participants’ coping self-efficacy and emotional coping. This positive finding for coping self-efficacy was also noted in two previously conducted studies: one testing a mind-body intervention for HIV-infected individuals [31] and another focusing on a self-directed cognitive behavioral therapy and mindfulness intervention for adults [32]. The previously conducted studies and our study included similar strategies to assist individuals in overcoming daily challenges to manage stress, such as becoming conscious of personal reactions to stress, drawing on personal strengths and abilities, taking responsibility for one’s health and well-being, and communicating in a positive and effective manner with others. The current study also showed a positive association between the intervention and emotional coping, which may have resulted from the intervention emphasizing the building of practical skills for coping with negative emotions (e.g., speaking positively to oneself, reflecting on one’s strengths, breathing deeply, counting to 10 to stay relaxed, and briefly removing oneself if possible from a stressful situation). Thus, researchers and health professionals interested in implementing an intervention to increase participants’ coping self-efficacy and emotional coping may want to include these strategies. We did not find any intervention effect on autonomous motivation. This unanticipated finding might have resulted due to low intervention adherence in the peer support group teleconference because of some scheduling difficulties between the participants and moderators who led the group [5]. Also, autonomous motivation was measured with items eliciting the degree that certain personal interests or values motivated the women to engage in health-promoting behaviors. The possibility exists that these specific items did not adequately reflect the personal interests or values of importance to the women in the current study. As a result, the measure may not have adequately captured the women’s autonomous motivation, even if their actual level of autonomous motivation was high.

The study findings indicated that higher autonomous motivation was associated with more depressive symptoms. This unexpected result is counter to research supporting that autonomous motivation contributes to enhancing positive mental health outcomes [16]. However, the current study’s finding is consistent with a prior study including elite team-sport athletes that showed a positive relationship between intrinsic motivation (the most autonomous form of motivation) and depressive symptoms [33]. One plausible explanation for this positive association is that increasing psychosocial demands experienced by individuals may result in more depressive symptoms that hinder improvement in autonomous motivation sufficient enough to curtail the rise in depressive symptoms [34]. Nevertheless, the findings highlight the complexities concerning the association between autonomous motivation and depression, meriting the need for further investigation. Consistent with findings from prior correlational research conducted with college age women [35], the current study showed a negative association between coping self-efficacy and the degree of depressive symptoms. The consistent findings regarding this negative association lend support for Bandura’s [19] propositions that self-efficacy influences how individuals persevere through difficult times, what self-regulative strategies they adopt to manage depression, and how vulnerable they are to depression; and that high self-efficacy can reduce depressive symptoms.

The current study showed no association between emotional coping and depressive symptoms. Emotional coping has been identified as an adaptive response that is helpful when individuals have no or little control over their situation [36]. Roth and Cohen [37] explain that emotional coping may be a preferred and appropriate approach when supportive resources are limited, the source of stress is unclear, or the problem is uncontrollable. However, it may not be beneficial for alleviating depressive symptoms in women having a low income who need to solve problems resulting from their low socioeconomic status, such as a compromised social network, chronic physical illness, or high risk for homelessness [38].

In terms of the mechanism for change, coping self-efficacy was the only psychosocial variable tested in the current study that mediated the association between the lifestyle behavior intervention and depressive symptoms. This finding was somewhat inconsistent with results from a prior study conducted by Silverstein and colleagues [39] that showed problem-focused coping did not mediate the relationship between a problem-solving educational intervention and depression among low-income mothers. Several possible factors may have contributed to the inconsistency. First, the current study used various strategies to improve skills for stress management, healthy eating, and physical activity, whereas the prior study only focused on increasing problem-solving skills [39]. Also, the present study included overweight or obese women who were predominately Non-Hispanic White women, while the prior research enrolled Non-Hispanic White with all body sizes [39]. The two groups may differ in their perceptions, depressive symptomatology, and response to an intervention. Moreover, the present study measured coping self-efficacy during a stressful situation, but the previous research measured coping self-efficacy as perceived control of life [39]. Regardless, the present study’s significant finding suggests the importance of focusing on coping self-efficacy in lifestyle behavior interventions to help low-income overweight or obese women with young children alleviate depressive symptoms. According to Gutierrez-Zotes [13], improving coping strategies through interventions is one of the most effective approaches for preventing depression in at-risk mothers after childbirth. The current study’s findings showed that neither autonomous motivation nor emotional coping mediated the association between the intervention and depression. Comparison of these findings to prior research was not feasible due to the lack of published studies examining these relationships.

### 4.1 Limitations

The current study has some limitations. The generalizability of the findings to women whose demographic characteristics differ from those included in the study sample may be limited. Relying on self-report for all measures can also be problematic because the women may have responded in a way that may not be indicative of their actual perceptions. Although the CESD is a validated measure for depressive symptoms [25], a clinician interview is the gold standard for diagnosing a mental health condition [22].

## 5. Conclusion

Of the three targeted psychosocial variables (autonomous motivation, coping self-efficacy, and emotional coping response), coping self-efficacy was the only one that mediated the association between the 16-week lifestyle behavior intervention and depressive symptoms in low-income overweight or obese mothers with young children. To more fully explain the intervention effect, continued testing of mechanisms that may underlie the alleviation of depressive symptoms from lifestyle behavior interventions implemented with this priority population is needed. This effort is important because insight into a lifestyle behavior intervention’s mechanism of action may lead to intervention refinements that, in turn, can help with simplifying intervention strategies and ultimately lead to effective dissemination and implementation.

## Figures and Tables

**Table 1:** Participant characteristics (N = 338: 212 intervention and 126 comparison).

	Intervention	Comparison	P-value
	Number (*%*) or Mean (*SD*)	Number (*%*) or Mean (*SD*)	
**Age (years)**	29.21 (4.92)	29.63 (4.95)	0.44
**Post-birth Time period (years)**	1.63 (1.23)	1.99 (1.30)	0.01
**Race**			0.08
Non-Hispanic White	179 (84.43%)	97 (76.98%)	
Non-Hispanic Black	33 (15.57%)	29 (23.02%)	
**Smoking**			0.28
Non-smoker	175 (82.55%)	98 (77.78%)	
Smoker	37 (17.45%)	28 (22.22%)	
**Education**			0.47
High school or less	59 (27.83%)	43 (34.12%)	
Some college or technical school	100 (47.17%)	54 (42.86%)	
College graduate or higher	53 (25.00%)	29 (23.02%)	
**Employment Status**			0.006
Full-time	34 (16.04%)	32 (25.40%)	
Part-time	46 (21.70%)	28 (22.22%)	
Unemployed	28 (13.21%)	28 (22.22%)	
Homemaker	79 (37.26%)	28 (22.22%)	
Self-employed, student and other	25 (11.79%)	10 (7.94%)	

**Table 2:** Direct and indirect effects of mediation testing while adjusting for baseline measures and covariates (N = 338: 212 intervention and 126 comparison).

	B (SE)	B (95% CI)	p-value	*β*	POMP
**Total Effect of Intervention**					
Intervention → Depressive symptoms_T2	−2.42 (1.00)	−4.37, −0.46	0.015	−0.11	−4.31%
**Direct Effects**					
Intervention → Autonomous motivation_T2	0.12 (0.08)	−0.04, 0.29	0.145	0.07	1.88%
Intervention → Coping self-efficacy_T2	0.30 (0.06)	0.19, 0.41	< 0.001	0.25	7.40%
Intervention → Emotional coping_T2	0.15 (0.05)	0.05, 0.24	0.003	0.18	3.81%
Autonomous motivation_T2 → Depressive symptoms_T2	1.70 (0.71)	0.30, 3.09	0.017	0.13	3.02%
Coping self-efficacy_T2 → Depressive symptoms_T2	−4.80 (1.37)	−7.48, −2.12	< 0.001	−0.25	−8.56%
Emotional coping_T2 → Depressive symptoms_T2	−1.77 (2.82)	−7.29, 3.76	0.53	−0.06	−3.15%
**Indirect Effects (Mediation)**					
Intervention → Autonomous motivation_T2 → Depressive symptoms_T2	0.21 (0.18)	−0.15, 0.57	0.256	0.01	0.37%
Intervention → Coping self-efficacy_T2 → Depressive symptoms_T2	−1.42 (0.46)	−2.33, −0.51	0.002	−0.06	−2.53%
Intervention → Emotional coping_T2 → Depressive symptoms_T2	−0.26 (0.43)	−1.10, 0.59	0.555	−0.01	−0.45%
**Direct Effect**					
Intervention → Depressive symptoms_T2	−0.95 (1.02)	−2.95, 1.06	0.355	−0.04	−1.68%
**Other Paths**					
Postpartum → Depressive symptoms_T2	0.48 (0.35)	−0.21, 1.17	0.171	0.06	0.85%
Depressive symptoms_T1 → Depressive symptoms_T2	0.61 (0.07)	0.47, 0.76	< 0.001	0.57	1.09%
Autonomous motivation_T1 → Autonomous motivation_T2	0.65 (0.07)	0.51, 0.78	< 0.001	0.65	9.90%
Coping self-efficacy_T1 → Coping self-efficacy_T2	0.79 (0.08)	0.64, 0.95	< 0.001	0.65	19.77%
Emotional coping_T1 → Emotional coping_T2	0.80 (0.09)	0.61, 0.98	< 0.001	0.84	20.94%

Note. B = unstandardized parameter estimate; *B* = standardized parameter estimate; SE = standard error; CI = confidence interval; POMP = proportion of maximum possible scores in the endogenous variable with per unit change in each exogenous variable; T1 = baseline; T2 = immediately after the 16-week intervention.
